# Pharmacist-Facilitated Interactive E-Learning for Patients Newly Initiated on Warfarin: A Randomised Controlled Study

**DOI:** 10.3390/pharmacy10010003

**Published:** 2021-12-23

**Authors:** Joanne Young, Michelle J. Nalder, Alexandra Gorelik, Rohan A. Elliott

**Affiliations:** 1 Pharmacy Department, The Royal Melbourne Hospital, Parkville 3052, Australia; Michelle.Nalder@mh.org.au; 2 Monash-Cabrini Department of Musculoskeletal Health and Clinical Epidemiology, Cabrini Health and Department of Epidemiology and Preventive Medicine, Monash University, Malvern 3144, Australia; alexandra.gorelik@gmail.com; 3 Department of Medicine (The Royal Melbourne Hospital), University of Melbourne, Parkville 3010, Australia; 4 Pharmacy Department, Austin Health, Heidelberg 3084, Australia; Rohan.Elliott@austin.org.au; 5 Centre for Medicine Use and Safety, Faculty of Pharmacy and Pharmaceutical Sciences, Monash University, Parkville 3052, Australia

**Keywords:** patient education, warfarin, anticoagulant, clinical pharmacy

## Abstract

It is not known whether electronic-learning (e-learning) is effective for educating hospital inpatients about complex medications such as warfarin. This prospective randomised controlled study compared pharmacist-facilitated e-learning with standard pharmacist-delivered face-to-face education on patients’ or their unpaid carers’ knowledge of warfarin and satisfaction with warfarin education as well as the time that was spent by pharmacists in delivering warfarin education. Adult English-speaking patients (or their carers) who had been prescribed warfarin were randomised to receive standard pharmacist face-to-face education (control) or an e-learning module on a tablet device facilitated by a pharmacist (intervention). All of the participants received written warfarin information and were presented with the opportunity to ask any questions that they may have had to a pharmacist. Fifty-four participants completed the study (27 per group). The participants who received e-learning had median correct Oral Anticoagulation Knowledge (OAK) test scores of 85% compared to 80% for standard education (*p* = 0.14). The participants in both groups were satisfied with the information that they received. There was a trend towards pharmacists spending less time on warfarin education for the e-learning group than in the standard education group (25.5 vs. 33 min, respectively, *p* = 0.05). Education delivered via pharmacist-facilitated e-learning was non-inferior in terms of patient or carer warfarin knowledge compared to standard pharmacist-delivered education.

## 1. Introduction

Warfarin is a complex, high risk medicine, with a low therapeutic index and potential for significant harm if used incorrectly. Warfarin education has been shown to be a significant predictive factor for bleeding complications, with increased bleeding rates being observed in patients who have been provided with insufficient warfarin education [1]. Inadequate education can influence anticoagulation control, with a positive correlation between patient warfarin knowledge and the number of International Normalised Ratio (INR) values within the target range [2]. Therefore, patient education regarding warfarin therapy is imperative to ensure its safe and effective use. 

A range of educational interventions aimed at improving patient knowledge of warfarin have been investigated in both observational [3,4,5,6,7] and controlled studies [8,9,10,11,12,13,14,15,16,17]. These have included face-to-face education from a healthcare professional (usually a pharmacist, nurse, or doctor) either delivered in a group setting [3], to individual patients [7,9,10], or via home visit after hospital discharge [15]. Some interventions have involved the addition of standardised information in a written format [4,11], in a video format [5,16], or a combination of both [8,17], whilst other interventions investigated the effect of a single teaching session using video only [6,12,13,14]. The results of the available evidence are inconclusive in terms of the most effective delivery mode. A review on the use of multimedia patient educational interventions on anticoagulation therapy highlighted the need for randomised clinical trials to assess the educational interventions and measure the outcomes of such studies using reliable, validated tools [18].

Interactive electronic-learning (e-learning) is an increasingly popular education delivery mode that has not been evaluated for its effectiveness in imparting warfarin knowledge to patients. E-learning typically consists of a combination of integrated video, animations, diagrams, pictures, and/or audio to deliver information electronically to patients in a structured manner [19]. Its benefits include the potential to increase the interest of learners through active participation in the learning process as well as increased information recall through the involvement of auditory, visual, and interactive learning strategies [19]. A Cochrane systematic review of evidence from 24 randomised controlled trials assessing multimedia-based medicine education programs suggests that this education delivery mode is at least equivalent to other forms of education, such as written education and education provided by a healthcare professional [20]. The effect of e-learning on patient knowledge has been investigated outside of the context of medicines. Randomised controlled studies that have been conducted in solid organ transplant recipients [21,22,23] and in adult patients with haemophilia on home treatment [24] demonstrated either no difference in knowledge [22,23] or an improvement in patient knowledge [21,24] when compared to standard care.

Interactive e-learning also has the capacity to allow for unlimited patient access, including after discharge from hospital, a desirable characteristic given that access to post-discharge education has been shown to significantly increase warfarin knowledge scores [25].

The primary objective of this study was to compare the effect of pharmacist-facilitated interactive e-learning versus pharmacist-delivered face-to-face education (standard care) on patients’ or their unpaid carers’ knowledge of warfarin. Secondary objectives were to compare the participants’ satisfaction with warfarin education as well as the amount of time spent by the pharmacist in delivering warfarin education, and to assess the preferences that pharmacists have in terms of the delivery mode for warfarin education. It was hypothesized that e-learning would be at least non-inferior compared to standard care in terms of patient knowledge but time-saving in terms of the amount of time spent by the pharmacist in delivering the education.

## 2. Materials and Methods

### 2.1. Design, Participants and Setting

This prospective, randomised controlled study was conducted between July 2015 and March 2016 at a public tertiary-care teaching hospital in Australia. Ethics approval was granted by the study site prior to commencement. Adult English-speaking hospital inpatients who commenced warfarin for any indication (or their carer, if planning to manage the patient’s warfarin administration) were eligible to participate. The term ‘carer’ in this study refers to unpaid carers, usually family members. Exclusion criteria included: cognitive impairment (documented diagnosis of dementia or positive MiniCog^©^ Test [26]); having previously been on warfarin within the last two years or, in cases where the carer was the study participant, if the carer had been prescribed warfarin within the last two years; pregnancy (for women); or if the participant was expected to be uncontactable via phone and either email or mail for the follow up assessment.

Patients (or their carers) from any hospital ward who had commenced warfarin during their inpatient stay were identified by ward clinical pharmacists. A member of the research team assessed eligibility, obtained written consent, and enrolled the participants.

### 2.2. Intervention and Standard Care 

Patients or carers who consented to participate were randomised to receive standard warfarin education from a pharmacist (control group) or warfarin education via e-learning (intervention group) facilitated by a pharmacist. Randomisation envelopes were prepared by an independent clinical trials pharmacist according to a block randomisation schedule that had previously been developed by the study statistician prior to study commencement, with fixed block sizes of two and four. 

Standard warfarin education reflected usual care and involved a pharmacist or provisionally registered (intern) pharmacist delivering verbal, face-to-face warfarin education, using a warfarin information booklet as a guide for the content delivered and answering patient (or carer) questions at the bedside prior to hospital discharge. At the study hospital, provisionally registered pharmacists were routinely permitted to provide warfarin education without direct pharmacist supervision once competency had been established. Once the provisionally registered pharmacists were assessed to be competent in delivering warfarin education to patients, their skills and knowledge for this activity were considered to be similar to those of a pharmacist. The term ‘pharmacist’ herein refers to either a pharmacist or provisionally registered pharmacist, unless otherwise specified.

The intervention group independently completed an interactive warfarin e-learning module with access that was facilitated by a pharmacist using an internet-enabled electronic device (either the participant’s personal device or a hospital tablet (iPad^®^)). This was followed by a face-to-face consultation with a pharmacist at the bedside prior to hospital discharge to answer questions and to address additional warfarin education needs. Pharmacists were trained on the study protocol and were aware that this participant interaction was not to repeat the information in the e-learning module but instead to identify any knowledge gaps. Written warfarin information was also provided (the same booklet that was provided to the control patients). The participants were able to re-access the e-learning module at any time, including after hospital discharge, if they had internet access and an electronic device, using an individual username and password.

The web-based e-learning module was developed with the assistance of an e-learning developer. The content was written by pharmacists at the study hospital in collaboration with medical and nursing staff and incorporated patient feedback that had been obtained according to hospital guidelines from five consumers using a written form. In terms of readability, the module aimed to obtain a Flesch–Kincaid Grade Level Score of seven or less (i.e., readable by those who have reached up to seventh grade of schooling) and incorporated best-practice recommendations for interactive, computer-based education described by Fox et al. [19]. An outline of the content is provided in Appendix A. The module’s interactive capabilities included navigating through pages, graphics, activities (e.g., select warfarin tablet strengths required for a 4 mg dose) and a 10-question knowledge quiz (different questions to those used to assess warfarin knowledge as the primary outcome). The module’s reporting function allowed the patient’s pharmacist to check whether the participant had completed the module and also gave them access the participant’s quiz score.

### 2.3. Outcomes

The primary outcome was participant warfarin knowledge at least two weeks after education, which was measured using the validated Oral Anticoagulation Knowledge (OAK) test [27]. The OAK test consisted of 20 multiple choice questions that were adapted to the Australian setting for the purposes of this study (e.g., Motrin^®^ changed to Nurofen^®^). The OAK test was emailed or posted (depending on their preference) to the participants on day 13, and answers were obtained between day 15 to 18 via a scripted phone interview that was conducted by a blinded research associate. The research associates who conducted the interviews were pharmacists at the study site who were not involved in providing care to the participants that they interviewed. Participant satisfaction with their specific warfarin education was measured via a phone survey that had been developed by the research team that included satisfaction statements that were answered by the participants using a five-point Likert scale. The survey also allowed the participants to provide comments. Answers were obtained during the phone interview, after OAK test completion. Responses were recorded in writing by the research associate. Interviews were not recorded.

The amount of time that the pharmacists spent providing warfarin education was self-measured and split into three components to reflect usual work-flow:

1. Providing initial education, such as a brief introduction to warfarin and/or providing the warfarin booklet to the patient/carer;
2. Providing standard or e-learning warfarin education (including opening the module on the device, if applicable) and answering patient/carer questions about warfarin;
3. Discharge education, which included explaining the dose, next INR, and answering final questions immediately prior to discharge.

All of the pharmacists who facilitated warfarin education via e-learning were provided with a paper survey that included an opportunity to indicate their preferred delivery mode. Survey completion was voluntary and implied consent for the information to be used as part of the study.

Statistical analysis was performed using Stata 12 (StataCorp. 2011. Stata Statistical Software: Release 12. StataCorp LP, College Station, TX, USA). A *p*-value of less than 0.05 was considered to be statistically significant. Between group differences in the OAK test scores (reported as percent answers correct) and the length of time that was spent by the pharmacists giving warfarin education were compared using the Wilcoxon Rank Sum Test. Comments that were provided in the surveys and during the interviews were analysed using thematic analysis by the principal investigator with input from the study team to identify themes. 

The estimated sample size for this study was 68 participants (34 per group), a number that was based on obtaining correct OAK test scores of 80% in the control group and a pre-specified non-inferiority margin of 10% in OAK scores, with a power of 80% and alpha = 0.05 while also allowing for a 20% drop-out rate. This sample size calculation and non-inferiority study design was based on previous research that had been conducted at the study hospital in 2014, which found that patients who began warfarin and were provided standard education by a pharmacist had a mean OAK test score of 80%, a value that was measured six weeks after education (unpublished).

## 3. Results

### 3.1. Participants

**Figure 1:**
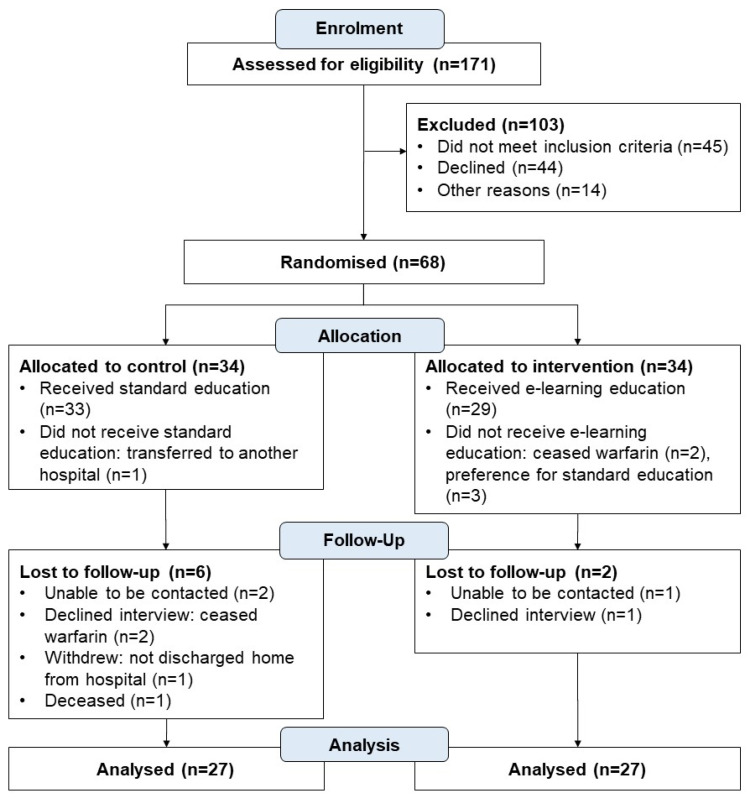
Study participant flow chart.

Of the 68 patients/carers who were enrolled and randomised, 54 participants (27 in each group) completed the follow up phone interview (OAK test and participant survey) and were included in the per protocol analysis (Figure 1).

Participants in the control and intervention groups were similar with respect to the baseline characteristics that were recorded (Table 1). However, a higher proportion of participants in the intervention group self-rated their confidence with electronic devices as ‘very confident and familiar’. The baseline characteristics of the participants that withdrew from the study or who were lost to follow up did not differ to those of the participants who were included in the analysis.

### 3.2. Outcomes

**Figure 2:**
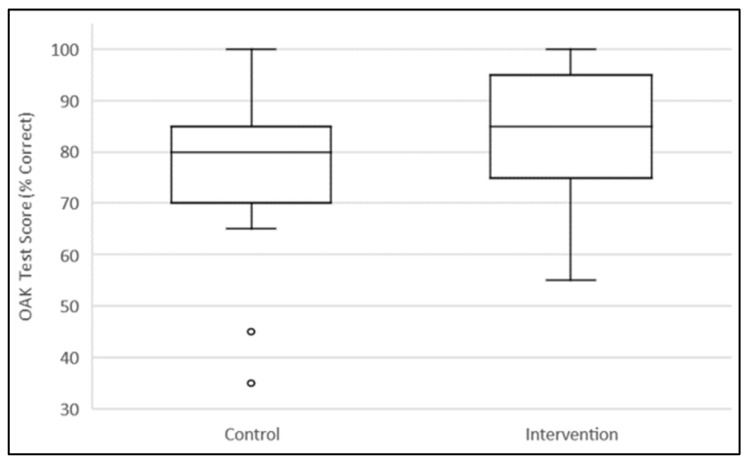
OAK test score box plot.

The median value of the correct OAK test scores in the e-learning group was 85% (interquartile range (IQR) 75 to 95) compared to 80% (IQR 70 to 85) in the standard education group (*p* = 0.14) (Figure 2). On average, the participants were tested 17 days after warfarin education. 

Sensitivity analysis was performed by excluding the three participants who had previously taken warfarin >2 years ago, which demonstrated similar results (median (IQR) OAK test scores of 85% (75–95%) and 82.5% (70–85%) for the intervention and control groups, respectively).

The participants in both groups were satisfied with the warfarin education that they received, with positive responses to each survey question being higher than 80% (Table 2). 

Comments received from control participants regarding standard pharmacist-delivered warfarin education were generally positive. Participants stated it was ‘great to have a face-to-face interaction’ and that they were impressed with the amount of detail and the way it was explained. Negative feedback related to the content included that it was ‘a lot of information, a lot to take in’; the inconvenient timing of education, such as during mealtime or when they were tired; that the pharmacist appeared to be rushed; or a preference for education earlier during the hospital stay. 

The intervention group participants also provided mainly positive comments. They stated that the e-learning was clear and easy to use, appreciated the ability to ‘log back on to do a refresh’, and valued being able to ask the pharmacist questions. Their negative feedback related to timing of education, being too tired resulting in them being ‘not able to take it all in’, and technological problems such as the Wi-Fi connection or being unable to finish the quiz ‘as the device wasn’t working properly’ (one participant). Many participants stated that e-learning may be best-suited to younger or ‘computer-savvy’ patients, whilst one participant acknowledged that ‘everyone might be able to use it, a lot of older people are now using iPads^®^’.

The pharmacists spent significantly less time facilitating participant access to the warfarin e-learning module for the intervention group than delivering standard face-to-face warfarin education for the control group (17 (IQR 10–25) vs. 25 (IQR 20–27) minutes; *p* = 0.03). However, there was no significant difference in the time spent by pharmacists providing an initial introduction to warfarin (4.5 (IQR 3–5) vs. 3 (IQR 2–5) minutes; *p* = 0.07) or in providing discharge warfarin education (5 (IQR 3–6) vs. 5 (IQR 3–10) minutes; *p* = 0.85). There was a trend towards a statistically significant difference between the groups in the total amount of time that was spent by each pharmacist on all aspects of warfarin education (25.5 (IQR 20.5–36) vs. 33 (IQR 30–35) minutes; *p* = 0.05). 

A total of 12 of the 13 pharmacists who enabled participant access to the e-learning module responded to the pharmacist survey (Table 3). Although the pharmacists found it easy to enable participant access to the e-learning module, unreliable hospital Wi-Fi and minor issues that occurred during participant login were raised. Only 4/12 pharmacists felt that they could identify gaps in participant knowledge following e-learning; qualitative feedback highlighted a desire to review which quiz questions the participants answered incorrectly to identify aspects of education to reinforce, and one response indicated difficulty navigating quiz responses. 

Nine out of twelve (75%) pharmacists reported that pharmacist-facilitated e-learning required less of their time compared to standard face-to-face warfarin education. Two (16.7%) pharmacists reported that education delivered via e-learning did not save time compared to standard education; one (who had facilitated the use of the module to a single participant) stated that the participant had not used an iPad^®^ before, whilst the other (who had also facilitated use of the module to a single participant) stated the participant had issues with their internet connection, thus requiring additional assistance. One (8.3%) pharmacist (who had facilitated use for three participants) provided a neutral response, as they felt that this was dependent on the participants’ familiarity with iPads^®^ or computers. 

Five out of twelve (41.7%) pharmacists indicated a preference for warfarin education delivered via e-learning. For one pharmacist (who had facilitated access to seven participants), this was a strong preference, whilst the three other pharmacists stated their preference applied to patients who are already able to use tablet devices or computers. Of the two (16.7%) pharmacists who indicated a preference for standard education, one had enabled access to a single participant and felt more confident in their ability to provide warfarin education but suggested using e-learning as a supplementary education tool. The second pharmacist had enabled access to eight participants during the study and expressed a preference to speak to patients directly in order to be confident that they understand the concepts. They stated that for ‘younger patients who quickly grasp the concepts, the e-learning module would be a good option’. Five (41.7%) pharmacists did not indicate a preference for warfarin education delivery mode due to only utilising e-learning with only one or two participants.

## 4. Discussion

Our findings support the hypothesis that warfarin education for hospital patients and/or their carers delivered via pharmacist-facilitated e-learning is non-inferior to standard pharmacist-delivered education in terms of imparting knowledge about warfarin. This indicates that interactive warfarin e-learning can be considered effective at delivering warfarin education to patients or their carers when the provision of written information and the opportunity to ask questions of a pharmacist at the bedside is maintained. 

Although direct oral anticoagulants are now used preferentially to warfarin for non-valvular atrial fibrillation (AF) [28], warfarin continues to play an important role in therapy for patients with valvular AF and for patients in whom direct oral anticoagulants are contraindicated, and its complexity and potential risk for harm means that the effectiveness of the strategies that are used for delivering warfarin education remains relevant to clinicians. Furthermore, our finding that pharmacist-facilitated e-learning was an effective education mode for a complex medication such as warfarin may be translatable, with further research, to other complex medications that require extensive patient education.

Our findings are consistent with results from previous studies focusing on interactive computer-based patient education programs about healthcare [19], multimedia-based education about medicines [20], and e-learning outside of the medicines context [21,22,23,24] that indicate that this innovative education strategy is at least as effective as the standard education that is provided by healthcare staff. Reasons why the e-learning module was able to effectively impart warfarin knowledge to participants may include that it was developed by practising pharmacists with extensive experience in delivering warfarin education, incorporated patient feedback during development, and that it was supplemented by a brief face-to-face pharmacist discussion to address participant questions and to reinforce key points. 

Although the pharmacists spent significantly less time facilitating warfarin e-learning compared to delivering standard face-to-face education, the study only demonstrated a trend towards a significant difference in the total time spent by pharmacists on all aspects of warfarin education. Possible reasons are the limited sample size, potential inaccuracy or bias in self-reporting of times, and lack of familiarity with facilitating patient access to the module. The rationale for maintaining significant pharmacist involvement in the education delivery process in the intervention group, rather than relying solely on the e-learning module, was due to the complexity of the information that patients must understand in order to use warfarin safely and the risks that are associated with warfarin misuse.

The feedback that was provided by the participants and pharmacists suggested that e-learning may not be suitable for everyone, such as some older patients or for those who are not familiar with using computers. It is likely that e-learning is also unsuitable for the types of patients who were excluded, such as those with cognitive impairment. An assessment of the educational needs of the patient or the carer and a determination of their learning preferences is essential to determine the most suitable mode of education. In addition, our study results were not impacted by participants previously taking warfarin, highlighting the importance of assessing individual patient learning needs despite prior warfarin therapy.

This study demonstrated higher OAK test scores in both the e-learning intervention (85%) and in the standard care (control) groups (80%) when compared to other studies that utilised the validated OAK test to assess the impact of their educational interventions on participants’ knowledge of warfarin [9,14,15]. In addition, this study also measured the amount of warfarin information that had been retained by the patients later on, at least two weeks following warfarin education. Correct OAK test scores in previous studies have ranged from 54.5% to 71.3% in the control groups and from 74% to 78% in the intervention groups [9,14,15]. Measurement times ranged from ‘at least 24 h after education but prior to discharge’ to day seven or day eight post education. The participants in the current study also had higher OAK test scores compared to those found in the OAK test validation study by Zeolla et al., which observed mean scores of 72% (±17.2) in patients who were already taking warfarin and 54% (±10.9) in warfarin naïve patients [27]. The current study’s higher OAK test scores observed were consistent with the previous (unpublished) research at the study site, in which mean OAK scores of 80% were observed six weeks following warfarin education delivered by a pharmacist using written warfarin information. 

This study did not measure improvements in knowledge from baseline for each participant; thus, it cannot be directly compared to previous observational studies [3,4,5,6,7]. A baseline knowledge test was not incorporated into the current study’s design to minimise any additional burden on participants who were expected to have low baseline knowledge, as they were all newly commenced on warfarin therapy. 

Previous controlled studies that have investigated warfarin educational interventions have demonstrated variability in their effect on participant knowledge [8,9,10,11,12,13,14,15,16,17]. The high effectiveness of warfarin education provided by pharmacists to the control group, as shown in the previously mentioned unpublished research at the study site, provided the basis for this study’s hypothesis of non-inferiority, which stated that the e-learning module would be as effective as standard education. Moore et al.’s 2013 study investigating video technology involved pharmacist-delivered education in a control group and found a similar result of there being no significant differences that were able to be observed in the OAK test scores [14]. Their study also detected a reduction in the time spent delivering education using a multimedia-based intervention (video) compared to usual care. 

The impact of providing written information on participant knowledge of warfarin in our study is unknown but would not have differed between study groups. The provision of written information was considered important to ensure patient safety and to meet the pharmacists’ professional obligations. At the study hospital, the pharmacist is the primary person responsible for educating patients about newly commenced medicines. Participants may also receive brief warfarin education from the hospital nursing or medical staff, and after discharge from their general practitioner, community pharmacist, and/or pathology clinic. 

Our study has some limitations. Firstly, the majority of the participants who were included were Caucasian, English-speaking and relatively young (mean age 55 years), which limits generalisability. Non-English-speaking patients/carers were excluded, as the e-learning was developed in English only. Secondly, the study was conducted at one inner-city hospital in Australia with a ward-based clinical pharmacy service. Results may not be generalisable to all other hospitals, especially those without clinical pharmacists providing patient education for patients commenced on new medicines. 

The target sample size for the study was not achieved due to a higher than anticipated drop-out rate, with 8.8% (6/68) of participants not receiving their allocated intervention due to ceasing warfarin, hospital transfer, or a preference for standard care (intervention group). This further highlights the need to consider patient preferences for education. A further 11.7% (8/68) of the participants were lost to follow-up, with reasons including declining the phone interview, being unable to be contacted, withdrawal, or death. However, the median correct OAK scores for the intervention group were higher than those of the control group, so it is unlikely that a larger sample size would have led to a finding of inferiority. 

Another limitation is potential selection bias. The study participants may have had a greater interest and desire to learn about warfarin, leading to a more engaged and motivated cohort. Additionally, those who were recruited may have had greater confidence with electronic tablet devices. A total of 22 of the 44 patients/carers who declined participation and who provided a reason for their refusal to participate indicated a preference for face-to-face education from a pharmacist, something that was often due to the technological aspect of the intervention. Patients/carers who declined participation tended to be older than those who participated (64 vs. 55 years). Participant health literacy was not measured, which could be a consideration for future research. 

A larger proportion of participants randomised into the intervention group reported higher self-rated confidence with electronic devices, which may have impacted the results. This, and the fact that patients who prefer face-to-face education sometimes declined to participate in the study, highlights the need to consider a patient’s individual preferences and familiarity with electronic devices (e.g., tablet device) when using e-learning in practice.

Blinding the participants and the pharmacists was not possible. The principal investigator and research associates, however, were blinded to study group allocation until after the OAK test and survey completion. There is the potential that the pharmacists modified their delivery of standard warfarin education because they were aware of the study, leading to a Hawthorne effect. However, the pharmacists at the study site were unfamiliar with the OAK test and were thus unlikely to have modified their education delivery to specifically target the test content. 

Lastly, this study focused on the effect of warfarin education on participant knowledge and satisfaction only and did not explore the effect on clinical outcomes such as time in therapeutic range and haemorrhagic or thromboembolic complications. The feasibility of obtaining INR results after hospital discharge prevented the inclusion of these outcomes in the study’s design. 

## 5. Conclusions

This is the first study that has investigated the effect of an interactive warfarin e-learning module on patient knowledge in the hospital setting. Our results support the use of pharmacist-facilitated e-learning to provide warfarin education to patients and carers. Although the patients and pharmacists were generally satisfied with this novel education delivery mode, it may not be appropriate for every patient and cannot completely replace pharmacist–patient interaction. It is important to maintain the opportunity for patients to ask questions and to be provided with written information. It is essential to determine the educational needs and learning preferences of the patient (or carer) in order to determine the most suitable education strategy. This study provides a basis for future research that explores the application of interactive e-learning modules for other medicines.

## Figures and Tables

**Table 1:** Baseline characteristics of study cohort.

Characteristic	Control n = 27 (%)	Intervention n = 27 (%)
Study participant type		
Patient	26 (96.3)	26 (96.3)
Carer	1 (3.7)	1 (3.7)
Age (years), mean (SD)	55 (14.2)	55 (15.6)
Gender (male)	18 (66.7)	20 (74.1)
English language spoken at home	25 (92.5)	26 (96.3)
Previously received warfarin therapy (>2 years ago)	1 (3.7)	2 (7.4)
Highest level of education achieved		
Tertiary	11 (40.7)	8 (29.6)
Secondary	10 (37)	14 (51.9)
Primary	6 (22.2)	5 (18.5)
No School	0	0
Self-rating of confidence with electronic devices (e.g., tablet device)		
Very confident and familiar	10 (37)	14 (51.9)
Somewhat confident and familiar	10 (37)	11 (40.7)
Used but not familiar	7 (25.9)	2 (7.4)
Not used	0	0
Access to electronic device after discharge	24 (88.9)	26 (96.3)
Access to internet after discharge	23 (85.2)	25 (92.6)

SD = standard deviation.

**Table 2:** Participant satisfaction survey responses.

Survey Statement	Control Group Responses n = 27 (%)			Intervention Group Responses n = 27 (%)		
	Positive ^1^	Neutral	Negative ^2^	Positive ^1^	Neutral	Negative ^2^
I am satisfied with the information I was given about warfarin	25 (92.6)	2 (7.4)	0	27 (100)	0	0
I am satisfied with the way warfarin information was presented to me	24 (88.9)	3 (11.1)	0	26 (96.3)	0	1 (3.7)
I am satisfied that the information provided was clear and easy to understand	23 (85.2)	3 (11.1)	1 (3.7)	25 (92.6)	2 (7.4)	0
I am satisfied that I had enough opportunity to ask questions about my warfarin therapy	25 (92.6)	1 (3.7)	1 (3.7)	23 (85.2)	2 (11.1)	2 (11.1)
Overall, I am satisfied with the manner in which the information was provided	24 (88.9)	3 (11.1)	0	25 (92.6)	2 (7.4)	0

^1^ Positive = Strongly agree/agree. ^2^ Negative = disagree/strongly disagree.

**Table 3:** Pharmacist survey responses.

Survey Statement	Pharmacist Responses n = 12 (%)		
	**Positive ^1^**	**Neutral**	**Negative ^2^**
I found it easy to enable study participant access to the interactive warfarin e-learning module	11 (91.7)	1 (8.3)	0
I considered the information delivered via the interactive warfarin e-learning module to be clear and easily understood by study participants	12 (100)	0	0
I found I could identify gaps in study participant knowledge following use of the interactive warfarin e-learning module	4 (33.3)	5 (41.7)	3 (25.0)
I found the interactive warfarin e-learning module took less of my time compared to when I deliver standard warfarin education	9 (75)	1 (8.3)	2 (16.7)
	**E-Learning Module**	**No Preference**	**Standard Education**
Overall preference for warfarin education delivery mode	5 (41.7)	5 (41.7)	2 (16.7)

^1^ Positive = Strongly agree/agree. ^2^ Negative = disagree/strongly disagree.

## Data Availability

The data presented in this study are available upon request from the corresponding author.

## Appendix A

**Warfarin E-Learning Module Content Outline**	
About this module Navigating this module What will I learn? What is warfarin? Why is warfarin used? What is an international normalised ratio (INR)? What will happen if my INR is not in my target range? What is an INR test and how do I get one? How often will I need an INR test? Can I choose a brand of warfarin? When should I take my dose? What time should I take my dose? How much warfarin do I take? Halving tablets How can I avoid missing a dose? What to do if I miss a dose?	What can affect my warfarin and/or INR? How do other medications affect my INR? Do I need to change my diet? How does health and lifestyle affect warfarin and/or my INR? Who needs to know I’m taking warfarin? Is it safe to become pregnant? Can I travel? Warfarin and bleeding Warfarin and serious bleeding How do I reduce my chance of bleeding? What are other side effects of warfarin? Will this affect my daily leisure activities? Key points to remember More information
